# Editorial: Treatment of comorbidities of asthma and its safety

**DOI:** 10.3389/fdsfr.2024.1366847

**Published:** 2024-02-05

**Authors:** Serghei Covantsev, Alexandru Corlateanu

**Affiliations:** ^1^ Department of Clinical Research and Development, Botkin Hospital, Moscow, Russia; ^2^ Department of Emergency Surgery N^º^76, Botkin Hospital, Moscow, Russia; ^3^ Department of Respiratory Medicine, Nicolae Testemitanu State University of Medicine and Pharmacy, Chișinău, Moldova

**Keywords:** asthma, commorbidity, spirometry, genetics, biomarkers, allergy

Comorbidity is defined as presence of two or more medical conditions in a patient. They tend to exist together for a number of reasons, such as common risk factors, etiology, pathogenesis or they are a part of the natural course of the disease. Asthma is the most widespread chronic respiratory disease worldwide that is often associated with multiple comorbidities. Approximately 300 million people have asthma worldwide and its incidence is likely to increase by another 100 million in the next years. Despite the fact that asthma is a chronic disease, it can lead to lethal outcomes and it is accountable for approximately 495 100 deaths in 2017 (Asher et al., 2020). Acute and chronic asthma complications are one of the common reasons for seeking medical help (Covantev et al., 2019). It is one of the leading causes of chronic morbidity at the global level and represents a major problem for public health. Taking into account these associations, the increase in the number of asthma patients will likely increase the hospital admission rate of patients with comorbidities, including exacerbation of asthma and other diseases.

**FIGURE 1:**
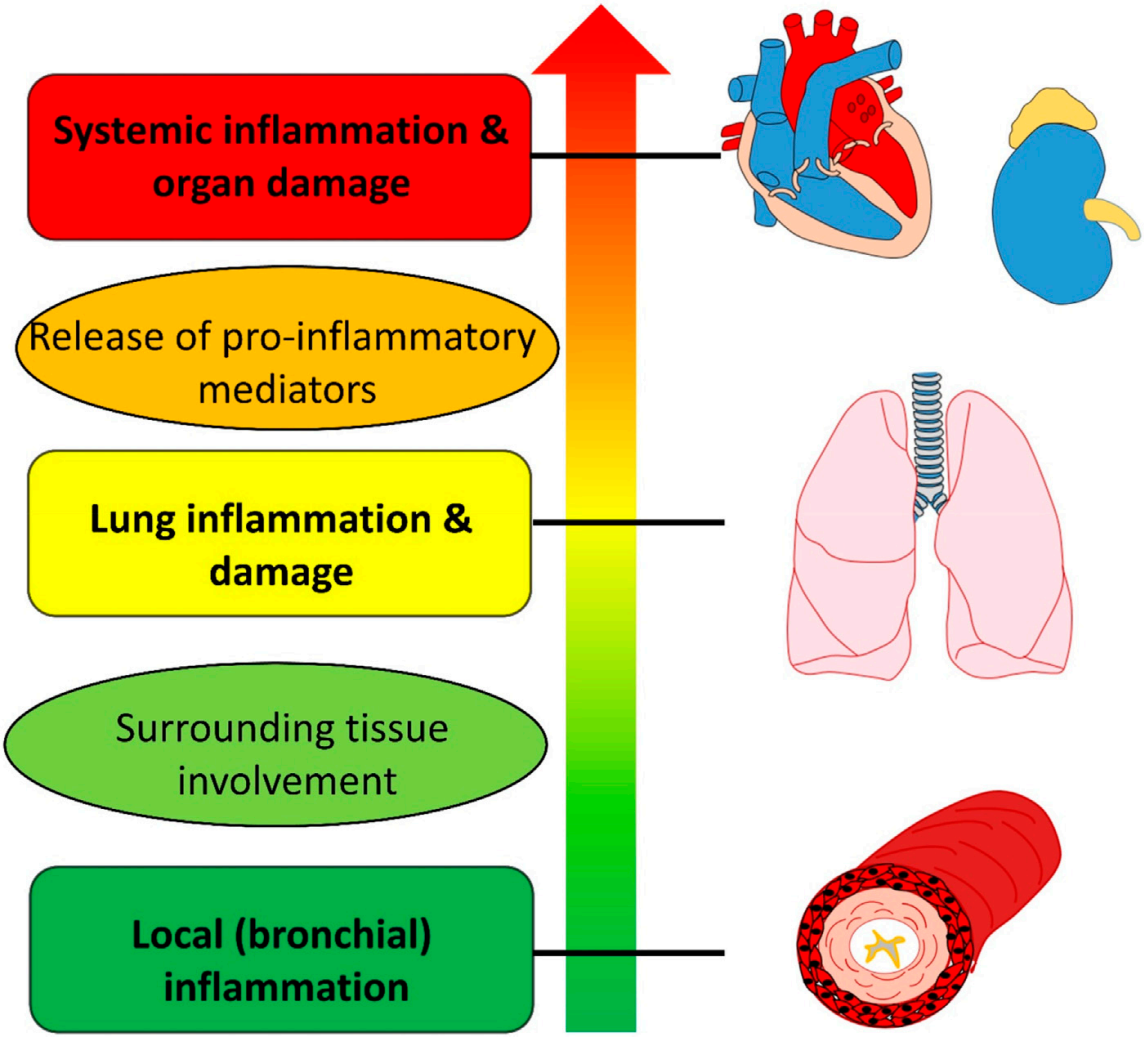
«Traffic light» stages of the inflammatory process (beginning as local inflammation in the bronchi and leading to systemic inflammation and organ damage).

Asthma is traditionally regarded as a lung disease, however, there has been substantial growth of evidence that it is an inflammatory disorder, which can expand beyond the lung tissue. The inflammatory process is primarily localized at the level of the bronchial tree. The inflammatory process can spread and involve the lungs and then lead to systemic inflammation via pro-inflammatory mediators, affecting other organ systems (Figure 1). Asthma leads to pulmonary and extrapulmonary comorbidities. The pulmonary complications of asthma include upper respiratory disorders such as allergic rhinitis, chronic rhinosinusitis, vocal cord dysfunction, obstructive sleep apnea syndrome and others. Asthma and allergic rhinitis are an example of systemic inflammation and a coexistence of two inflammatory diseases that lead to disability, as demonstrated in the AROCAT. The study investigated asthma comorbidities in six different hospitals in Thailand and included 682 asthma patients and demonstrated allergic rhinitis associated with asthma control, quality of life, and pulmonary function Sriprasart et al. Lower respiratory disorders that tend to coexist with asthma are COPD and bronchiectasis. COPD is a condition that can overlap with asthma, therefore increasing respiratory load. As it was demonstrated in the AROCAT study, lung ventilation in patients with comorbidities tends to decrease, which in turn can also lead to COPD Sriprasart et al. Extrapulmonary comorbidities include practically every system of the body: cardiovascular, gastrointestinal, metabolic, nervous etc. Asthma is a major risk factor of myocardial infarction, stroke, lower respiratory infections, tracheobronchial cancer, diabetes mellitus, chronic kidney disease and other conditions that are listed among the “top 10 causes of death” by World Health Organization (Corlateanu et al., 2021; WHO, 2024).

Asthma diagnosis and treatment depends on proper standardization of healthcare products. There have been attempts to standardize healthcare product using specific directives and regulations. However, qualification issues may arise for medical devices and their accessories, medicinal products. The regulatory status of gases for spirometry is discussed in a separate article by Manellari et al.

It is important to assess the asthma comorbidome holistically as it provides valuable information to a caregiver and proper management improves quality of life and outcomes in asthma. Profile of difficult to treat asthma patients is associated with multiple comorbidities that require systematic assessment (Radhakrishna et al., 2016). Treatment of comorbidities appears to improve asthma outcomes (Porsbjerg and Menzies-Gow, 2017). Today, the use of precision medicine guided by biomarkers offers new perspectives on asthma management. Stratification of asthma into phenotypes can be based on clinical features, genetics, proteomics, metabolomics analysis and biomarkers. Bonnesen et al. provide a review of T2-high, T2-low, obesity-associated asthma and frequent exacerbators. The manuscript overviews more than 50 potential biomarkers that can be helpful for asthma assessment. The latter is a promising field as it allows personalized treatment based on the key pathogenesis. Identifying biomarkers that may reveal underlying disease pathophysiology can help select patients who will benefit most from specific treatments.

Finally, some of the asthma comorbidities may be genetically predicted. The study by Wang et al. suggest a significant association of COPD, chronic sinusitis, atopic dermatitis, allergic conjunctivitis and allergic rhinitis with asthma and a potential higher risk of allergic urticaria

The goal of the current Research Topic is to overview comorbid conditions that are associated with asthma and to assess the safety of current medications and the interference of asthma drugs with comorbidities.
